# In vitro evaluation of the color stability and surface roughness of a new composite flow

**DOI:** 10.4317/jced.60005

**Published:** 2023-01-01

**Authors:** Gina Khazaal, Maha Daou, Syed-Sarosh Mahdi, Zohaib Ahmed, Elie Maalouf, Gopi Batteni, Syed-Saad B. Qasim, Cynthia Kassis, Daniyal Agha, Helene Haddad, Ryan el Hajj Assaf, Carina-Mehanna Zogheib

**Affiliations:** 1 Department of Esthetic and Prosthetic Dentistry, Dental Faculty, Saint Joseph University of Beirut, Lebanon; 2 Director of Postgraduate Program in Research and Dental Materials, Department of Pediatric Dentistry, Dental Faculty, Saint Joseph University of Beirut, Lebanon; 3 Dental Section Division of Clinical Oral Health Sciences, School of Dentistry, International Medical University, Kuala Lumpur, Malaysia; 4 Centre of Clinical Research, Telemedicine and Telepharmacy, School of Medicinal and Health Products Sciences, University of Camerino, 62032 Camerino, Italy; 5 Athena Center for Advanced Research in Healthcare, 62032 Camerino, Italy; 6 Research member-New York Chapter NYHDA, USA. College of Dental Medicine, Columbia University, NY, USA; 7 School of Dental Medicine, Lebanese University, Lebanon; 8 Department of Bio Clinical Sciences, Faculty of Dentistry, Kuwait University, Kuwait City 12037, Kuwait; 9 Dental Faculty, Saint Joseph University of Beirut, Lebanon; 10 Dental Section Jinnah Medical and Dental College, Sohail University, Karachi. 74800, Pakistan; 11 Dr Ryan Assaf’s Private Dental Clinic, Beirut Lebanon; 12 Director of Postgraduate Program in Restorative and Esthetic Dentistry, Dental Faculty, Saint Joseph University of Beirut, Lebanon

## Abstract

**Background:**

The aim of this study was to evaluate the color stability and the surface roughness of a bulk-fill composite flow (SDR® Plus) by comparison to an ORMOCER-based composite (Ceram.x® Universal SphereTEC™) in order to confirm the validity of using SDR® Plus in the anterior region and to allow the prediction of its long-term results.

**Material and Methods:**

35 composite specimens of the same shade (A2), thickness (2mm) and shape of both types of composite were prepared. The specimens were cured and polished according to the manufacturer’s instructions. The initial shade of the specimens was measured using a calibrated EasyShade spectrophotometer. The initial surface roughness of the specimens was measured by AFM. Afterwards, the specimens were subjected to an accelerated aging procedure through thermo-cycling, a coffee stain challenge and brushing to simulate two years in the oral environment. The shade and surface roughness of the specimens were measured again after the accelerated aging procedure.

**Results:**

The mean ΔE was significantly larger than 3.368 in Ceram.x® group (-*p*-value<0.001) and SDR® Plus group (-*p*-value<0.001). The mean surface roughness has significantly increased for both groups after aging with no significant difference between the two groups. It however remained clinically acceptable.

**Conclusions:**

SDR® Plus and Ceram.x® showed similar surface roughness when subjected to the same testing conditions. Concerning the color stability, both composites displayed noticeable discoloration, with higher ΔE values registered for Ceram.x®.

** Key words:**Composite resins, spectrophotometry, atomic force microscopy, dental material, resin-based material.

## Introduction

Resin Based Composite (RBC) filling materials are nowadays the most commonly used products in conservative restorative dentistry (1). Over the years, the longevity of RBC restorations has been improved by enhancing their bonding to enamel and dentin (2). Likewise, the properties of the fillers and the chemical structure of the monomers have been modified to influence different physical properties. Knowing that when one property prevails, another might be undermined, different categories of composite emerged to fulfill the requirements of the different clinical situations.

In order to provide good marginal adaptation and reduce the polymerization stress, low viscosity bulk-fill composites were produced and it became possible to build up a 4mm composite layer in one increment reducing working time, and providing homogenous restorations through a simple technique. Though these low viscosity bulk-fill composites are very practical, their esthetic and mechanical properties are not sufficient to place them on the surface; they have to be covered with a layer of a medium viscosity conventional composite. As a consequence, products belonging to this category of composite (such as Surefil® SDR™flow, Dentsply, Caulk, USA) are preferably used for the direct restoration of deep cavities in posterior teeth (3). Currently SDR® flow+ has been developed with improved wear resistance and the availability of four shades. Therefore it can be placed without an additional capping layer and the indications of this low viscosity bulk-fill composite are widened to include the restoration of class III and V cavities (4).

Applying a low viscosity bulk-fill composite in the anterior area is very much at the cutting-edge. However, in order to place a composite in surface, it must fulfill multiple criteria such as high resistance to abrasion, high wear resistance, hardness, traction resistance, shade stability, gloss and low surface roughness after finishing and polishing (5). In this perspective, it would be interesting to compare the esthetic properties of SDR flow+ to that of an esthetic composite that already proved itself in clinical practice (6) such as Ceram.x® Universal SphereTEC™ (Dentsply, Caulk, USA).

The purpose of this study is to evaluate the color stability and the surface roughness of SDR® flow+ by comparison to Ceram.x® (both issued by the same manufacturer) in order to confirm the validity of using SDR® flow+ in the anterior region and to allow the prediction of its long-term results. The null hypothesis is that no significant difference will be found between the color stability and surface roughness of SDR® flow+ and Ceram.x® when subjected to the same testing conditions.

## Material and Methods

This study compares the shade stability and surface roughness of SDR® Plus and Ceram.x® Universal SphereTEC™ (an esthetic composite that already demonstrated appropriate shade stability and surface roughness).

-Specimen preparation

Thirty-five rectangular samples (8 mm length x 4 mm width x 2 mm depth) were made of A2 shade of each composite. The composite was injected into a mold, covered with a Mylar strip and gently pressed with a microscope glass to eliminate the excess material and insure a homogenous surface. Each specimen was polymerized for 20 seconds using a LED light with a wavelength of 430-480 nm and a light intensity of 1000-1200 mW/cm2 (Woodpecker DTE Curing Light LUX.VI), as recommended by the manufacturer.

All specimens were finished by the same operator using Enhance® finishing points (Dentsply Caulk, Milford, DE, USA) for 20 seconds, then polished using Enhance® polishing cups and Prisma® GlossTM Composite Polishing Paste (Dentsply Caulk, Milford, DE, USA) for 20 seconds with a micro-motor at low speed. To remove debris, the specimens were rinsed for 10 seconds and air dried for 5 seconds.

-Simulated aging procedures

In order to predict long term results in conditions similar to those of the oral cavity, the composite specimens must be exposed to accelerated aging simulating procedures affecting their shade stability and surface roughness.

The specimens of each group were aged by thermo-cycling and light exposition according to the ISO norm 7491:2000 for dental materials and then determination of color stability was done. To simulate 2 years of in-vivo exposure to the oral environment, the specimens were exposed to 20000 cycles in the SD Mechatronik Thermocycler (SD Mechatronik GmbH, Feldkirchen-Westerham, Germany) alternating between 5ºC and 55ºC water baths with 20 seconds dwell time and a 10 seconds rest time.

The specimens were afterwards exposed to a thermo-cycling stain challenge, the purpose being a realistic simulation of clinical exposure of the composite to staining agents that would influence its shade stability. Assuming the composite restoration would be exposed to a staining agent such as coffee for about 15 minutes per day in-vivo (1 or 2 cups per day), the specimens were exposed to 200 cycles in the SD Mechatronik Thermocycler alternating between a 5ºC water bath and 55ºC coffee bath with 20 seconds dwell time and a 10 seconds rest time.

**Figure 1:**
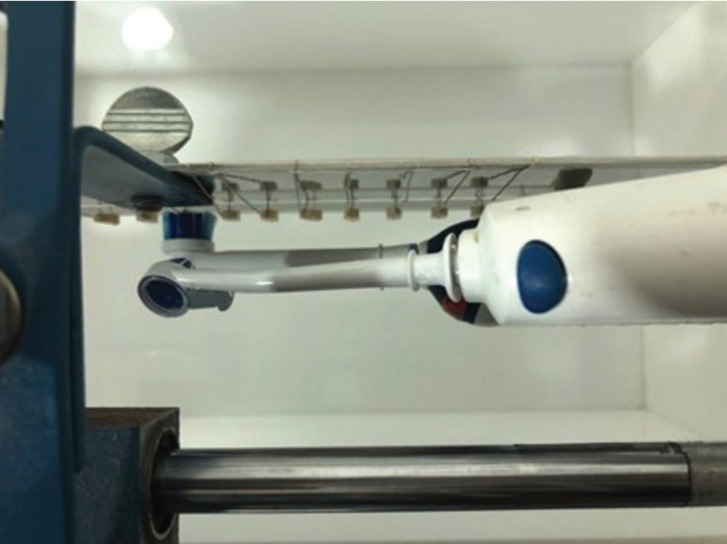
Brushing of the composite specimens.

Following this procedure, the specimens were rinsed and left in artificial saliva. To recreate the effect of toothbrush abrasion, that would affect the surface roughness of composite restorations, a tooth-brushing simulator was used. Specimens were fixed on a holder while an electrical toothbrush (Oral-B Professional Care OxyJet +3000, Braun, Frankfurt, Germany) went back and forth brushing them with an emission of toothpaste and artificial saliva (with a paste-to-saliva ratio of 1:2) at a standardized pressure of 2 N. The brushing speed is equivalent to 59.5 rpm (119 strokes per minute). Each brushing cycle consists of a back and forth motion (2 strokes). To simulate a month of brushing (Fig. 1), each specimen must be subjected to 833 strokes or 7 minutes of simulated brushing. Ultimately, each specimen was subjected to 168 minutes of brushing since this in-vitro study aims to mimic 2 years of clinical testing.

The specimens were then rinsed and well dried in order to proceed with the shade and surface roughness measurements.

-Shade measurements

The initial shade of the specimens was measured directly after their preparation. It was measured again after the accelerated aging process to allow the assessment of shade stability. The shade measurements were realized by the same operator using a calibrated EasyShade spectrophotometer (VITA Zahnfabrik, Bad Säckingen, Germany) and a neutral grey background.

The registered CIE L*a*b* values obtained with the EasyShade spectrophotometer allow the calculation of the difference in the general shade (ΔE) of each specimen and the statistical comparison of the color stability between both groups and among each group thus allowing clinically relevant shade monitoring.

-Surface roughness evaluation

**Figure 2:**
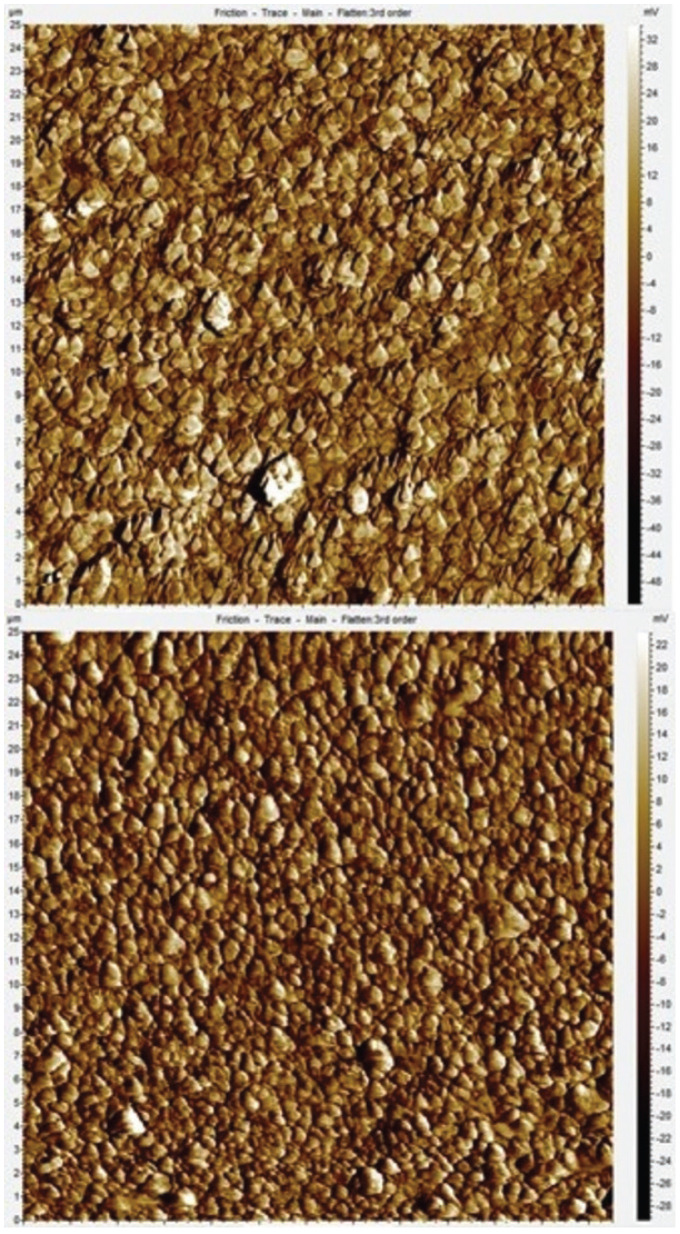
Atomic Force Microscope Image comparision - Ceram.x® initial surface and after artificial aging.

**Figure 3:**
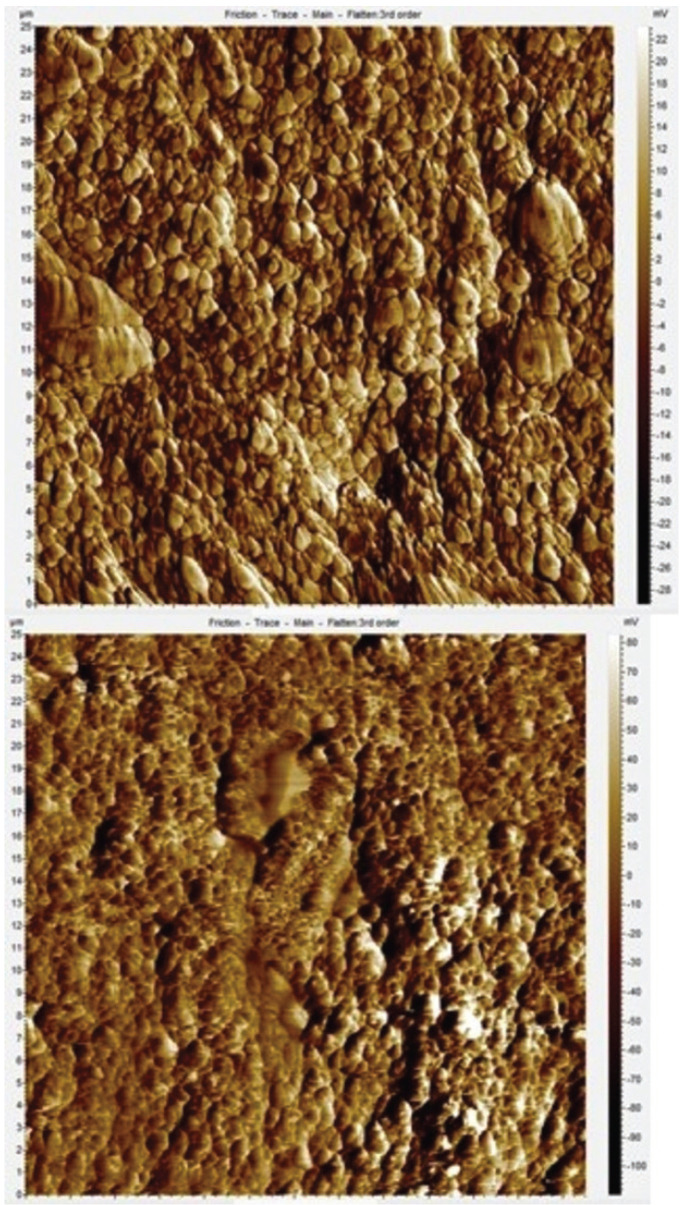
Atomic Force Microscope Image comparision - SDR® Plus initial surface and after artificial aging.

The average surface roughness (Ra) was measured before and after the aging of the specimens with the Agilent Technologies 5420 Atomic Force Microscope (AFM) in contact mode, using cantilevers with a constant spring of 0.6 N/m and CSG30 NT-MDT Tips. For each specimen three images of 25x25 nm were acquired with a resolution of 512x512 pixels. The images were analyzed using the PicoView 1.14 Software (Agilent Technologies) and the Ra of each specimen was the mean result of the Ra gathered from the three images (Figs. 2,3).

-Statistical analysis

The statistical analyses were performed using IBM SPSS Statistics (version 26.0). The level of significance was set at -*p-value* ≤0.05. The primary outcome variable of this study was the ΔE.

Kolmogorov-Smirnov tests were used to assess the normal distribution of quantitative variable among groups. Parametric tests were used for variables normally distributed. Non-parametric tests were also used for variables not normally distributed. Independent Student t tests and Mann-Whitney tests were used to compare continuous variables (ΔE, a, b, L) between Ceram.x® and SDR® Plus.

The 95% confidence interval of the mean ΔE values was calculated in each group. One-Sample t tests were used to compare the mean ΔE with 1.2, 2.767 and 3.368 threshold. Paired Student t tests and Wilcoxon tests were used to compare ΔL, Δa and Δb before and after artificial aging. Repeated-measure analysis of variance followed with univariates analyses were used to compare surface roughness variation throughout time and among groups.

## Results

-Comparison of ΔE among groups

The mean, standard-deviation, minimum and maximum of the ΔE in each group are displayed in the following Table 1. This study revealed that the mean ΔE value was significantly different between Ceram.x® and SDR® Plus groups (-*p-value*<0.001); the mean ΔE was superior in Ceram.x® group in comparison to SDR® Plus.

**Table 1:**
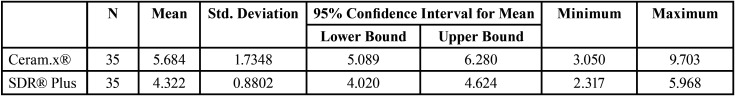
Mean ΔE among groups.

One-Sample t test was used to compare the mean ΔE value with the acceptability threshold of 3.368; the results showed that the mean ΔE was significantly larger to 3.368 in Ceram.x® group (-*p-value*<0.001) and SDR® Plus group (-*p-value*<0.001).

-Comparison of color component among groups

The mean and standard-deviation of each component of color in each group are described in the following Table 2.

**Table 2:**
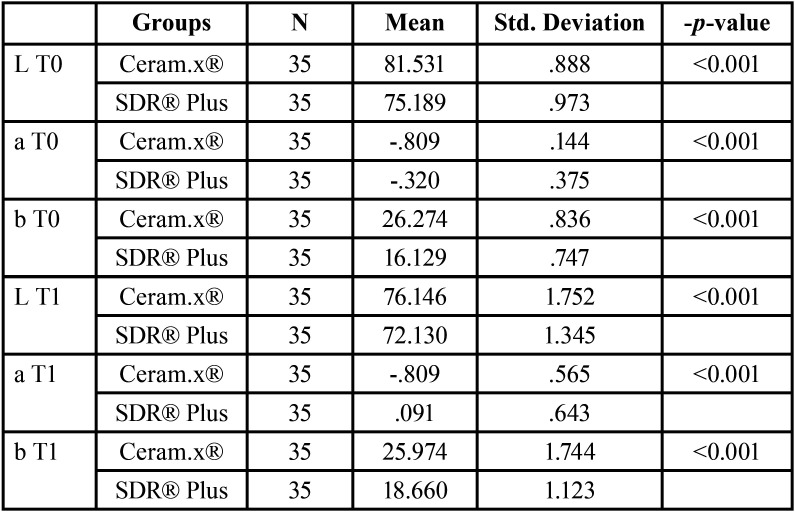
Mean values of each color component among groups.

The mean “L” values at T0 and T1 were significantly greater in Ceram.x® group compared to SDR® Plus (-*p-value*<0.001).

The mean “a” values at T0 and T1 were significantly greater in Ceram.x® group compared to SDR® Plus (-*p-value*<0.001).

The mean “b” values at T0 and T1 were significantly greater in Ceram.x® group compared to SDR® Plus (-*p-value*<0.001).

For Ceram.x®:

• The mean L value has significantly decreased between T0 and T1 (-*p-value*<0.001).

• The mean a value has not significantly changed between T0 and T1 (-*p-value*=1.000).

• The mean b value has not significantly changed between T0 and T1 (-*p-value*=0.292).

For SDR® Plus: 

• The mean L value has significantly decreased between T0 and T1 (-*p-value*<0.001).

• The mean a value has not significantly changed between T0 and T1 (-*p-value*=1.000).

• The mean b value has significantly increased between T0 and T1 (-*p-value*<0.001).

-Comparison of the surface roughness among groups

The mean and Standard deviation of the surface roughness in Ceram.x® and SDR® Plus groups at baseline and T1 are displayed in the following Table.

• Comparison throughout time

The mean surface roughness has significantly increased between baseline and T1 for Ceram.x® group (-*p-value*=0.004).

Similarly, the mean surface roughness has significantly increased between baseline and T1 for SDR® Plus group (-*p-value*=0.005).

• Comparison between Ceram.x® and SDR® Plus

Our results revealed that the mean surface roughness was not significantly different between Ceram.x® and SDR® Plus at baseline (-*p-value*=0.319) and T1 (-*p-value*=0.367).

Moreover, the increase in surface roughness between T0 and T1 was not significantly different between Ceram.x® and SDR® Plus (-*p-value*=0.946; Statistical interaction).

## Discussion

In this study, each RBC specimen was prepared using a Mylar strip to promote smooth surfaces (7). Afterwards, an adequate polishing procedure was realized to ensure the surfaces initially present maximum chromatic stability, as demonstrated by Macedo *et al*. (8) as well as a clinically ideal surface roughness (Ra < 0.2 µm) related to a favorable surface gloss and a minimal risk of plaque adhesion to the RBC (9).

The oral environment imposes many chemical and physical constraints on RBCs (10). However, the initial screening of dental materials occurs through in-vitro tests. Although thermo-cycling is the most commonly used artificial aging technique, it unfortunately lacks a standardized protocol across studies (11). The thermo-cycling protocol based on Gale and Darvell’s proposition, that 10 000 thermal cycles with temperatures alternating between 5°C and 55°C represents nearly one year of in-vivo functioning, (12) was adopted in this study, and the specimens underwent 20 000 thermal cycles to simulate two years in-vivo.

Coloring substances found in the daily diet can be absorbed or adsorbed by RBCs. The use of a coffee solution during the artificial aging procedure can be a highly effective stain challenge to the RBCs seeing that coffee presents a yellow-stain molecule that can be compatible with the resin polymer chain. This could favor the adsorption and penetration of the coffee dye into composites, which is in agreement with studies showing that coffee solutions showed the highest ΔE values while studying the color stability of RBCs (8,10,13).

Different staining simulation protocols can be found in the literature, nevertheless, thermo-cycling intercalated baths in staining beverages were formulated in order to simulate a more realistic clinical situation since patients are in contact with the colorants in intercalated periods (8).

Enamel, dentin and RBC restorations are highly affected by tooth brushing. Therefore, it is recommended to incorporate tooth brushing into in-vitro study designs involving discoloration evaluation (14). In fact, the simulated brushing not only tests the material’s ability to maintain its shine but also affects the surface roughness of RBCs, which in turn promotes higher light refraction indexes leading to darker color registrations. However this effect varies depending on the RBC composition (10). The brushing model established by Sexson and Phillips was consequently used in this study (15).

The shade measurements were realized using the Easy Shade spectrophotometer that was proven to be reliable and precise in the color measurement of dental materials (16). A neutral grey background was used for that matter to avoid controversies, since the use of white backgrounds is highly reflective and doesn’t seem to relate to the clinical situation of anterior teeth that are contrasted by a dark background, however the use of a black background may present discrepant spectrophotometric readings (17).

Color differences (ΔE*ab) can be calculated using the color coordinates gathered with Easy Shade spectrophotometer. In dental research, a quantitative representation of the color difference can be achieved through the application of various color difference formulas. The most frequently used formula derives from the CIE L*a*b* system (18) (4):

**Figure 4:**
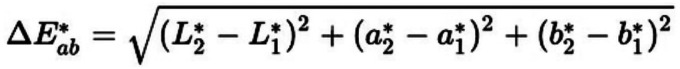
Formula.

The 50:50 perceptibility threshold refers to a color difference perceived by 50% of observers (the other 50% will notice no difference between the compared colors). While the 50:50 acceptability threshold refers to a color difference considered accepTable by 50% of observers while the other 50% consider it unacceptable (19).

The literature review conducted by Khashayar *et al*. cited different studies that reported ΔE*ab thresholds for clinically perceptible and acceptable color differences (18). In fact, ΔE*ab = 1.2 is frequently considered the 50:50 perceptibility threshold when observing opaque monochromatic samples under controlled conditions (only 50% of observers will notice the color difference between compared objects). Whereas the 50:50 acceptability thresholds in the oral cavity is between a ΔE*ab of 2.767 and 3.368 (50% of observers will accept the restoration and 50% will have it replaced because of color mismatch). However, there is a wide array of findings on visual color thresholds reported in several studies, more or less controlled (20,21).

Although all specimens are of the same shade, the initial shade measurements showed a slight variability in the registered values for each specimen within both groups. This variability could be due to the fact that for each specimen an individual composite capsule was used.

According to the results of this study, both composite groups suffered significant discoloration with ΔE > 3.368, exceeding the threshold of acceptability. However, SDR® Plus demonstrated less discoloration with inferior ΔE and L, a, b values compared to Ceram.x®. Concerning the color components, the results indicate a significant decrease in lightness (L values) in both composite groups after artificial aging. Additionally, SDR® Plus displayed significantly increased yellowness (b values) compared to its initial shade.These outcomes are in concordance with the results of Poggio *et al*. and Silva *et al*. that denote the particular effect of coffee on the color stability of modern esthetic RBCs (13,22). The difference in discoloration between the two types of RBCs can be traced back to the difference in their composition and water sorption tendency. In the study conducted by Salgado *et al*. the composites with higher inorganic content ratio by weight manifested the higher color differences (23).

The evaluation of the surface roughness of the specimens was conducted through the use of AFM due to its superior capability to distinguish surface roughness by comparison to Profilometry, and its higher definition of the surface by comparison to SEM (24). The AFM measurements revealed no significant difference between the initial surface roughness of Ceram.x® and SDR® Plus. Both composite groups exhibited significant increase in surface roughness after the simulated aging procedure, nevertheless both groups still had Ra < 0.2 µm. This proves both composites are equally convenient for placement in surface. These findings are in agreement with the study conducted by O’Neill *et al*. in which SDR® Plus presented an increased mean surface roughness that remained lower than 0.2 µm after a similar brushing protocol (24).

Seeing that this is an in-vitro study, the oral environment conditions aren’t entirely replicated regarding the presence of enzymes, proteins, pH variations, flexural constraints. These factors, among others, play a role in the intrinsic and extrinsic discoloration of teeth with aging (25). A correlation between the variations enamel and dentin would undergo, if subjected to this protocol, and the variations the RBCs underwent would be interesting and may allow better judgment of the RBCs clinical behavior. In fact, it has been established that discoloration of dental hard tissue and RBC restoration mismatch is affected by both the staining agent in question and the type of RBC (26,27). For that matter, enamel is weakened and decalcified by the erosive effect of coffee, which facilitates coffee staining on teeth (28). However the variation in enamel surface roughness alone doesn’t seem to affect the overall color of the tooth (29).

Consequently, to dental hard tissue being also subjected to discoloration, the tolerance for shade match perceptibility and acceptability may be higher in-vivo than in-vitro. Additionally, as patients tend to have higher perceptibility and acceptability thresholds than dentists, the color changes detected in the RBCs may need to be further investigated clinically and with the patient’s perspective taken into account (30). Another clinically relevant factor to take into consideration is the increment thickness of the bulk-fill composite. Although in this study all specimens were 2 mm thick, it has been demonstrated that bulk-fill materials may present lower depth of cure when placed in thicker increments which could lead to greater staining susceptibility (31). Moreover, composite discoloration due to exposure to staining beverages can be significantly reduced through re-polishing, which can render the restorations clinically acceptable once again (32).

## Conclusions

The null hypothesis suggested can be partially accepted: concerning the surface roughness, SDR® Plus and Ceram.x® showed similar and satisfying behaviors when subjected to the same testing conditions. Concerning the color stability, both composites displayed noticeable discoloration, with higher ΔE values registered for Ceram.x®. Within the limitations of this study, it can be concluded that SDR® Plus can be used in the anterior region just as well as Ceram.x®. However, further clinical observations will be needed to confirm these results.
